# Critical illness among patients experiencing homelessness: a retrospective cohort study

**DOI:** 10.1186/s13054-023-04753-7

**Published:** 2023-12-06

**Authors:** K. M. Sauro, C. M. O’Rielly, J. Kersen, A. Soo, S. M. Bagshaw, H. T. Stelfox

**Affiliations:** 1 Departments of Community Health Sciences, Surgery and Oncology, O’Brien Institute for Public Health and Arnie Charbonneau Cancer Institute, Cumming School of Medicine, University of Calgary, 3280 Hospital Dr. NW, Room 3D41, Calgary, AB T2N 4Z6 Canada; 2 Cumming School of Medicine, Calgary, AB Canada; 3 Department of Community Health Sciences and O’Brien Institute for Public Health, Cumming School of Medicine, University of Calgary, Calgary, AB Canada; 4 Department of Critical Care Medicine, Cumming School of Medicine, University of Calgary, Calgary, AB Canada; 5 Department of Critical Care Medicine, Faculty of Medicine and Dentistry, University of Alberta, and Alberta Health Services, Edmonton, AB Canada; 6 Critical Care Strategic Clinical Network, Alberta Health Services, Alberta, Canada

**Keywords:** Homeless, Healthcare resource utilization, Equity, Health services, Vulnerable population

## Abstract

**Purpose:**

To understand the epidemiology and healthcare use of critically ill patients experiencing homelessness compared to critically ill patients with stable housing.

**Methods:**

This retrospective population-based cohort study included adults admitted to any ICU in Alberta, Canada, for a 3-year period. Administrative and clinical data from the hospital, ICU and emergency department were used to examine healthcare resource use (processes of care, ICU and hospital length of stay, hospital readmission and emergency room visits). Regression was used to quantify differences in healthcare use by housing status.

**Results:**

2.3% (*n* = 1086) of patients admitted to the ICU were experiencing homelessness; these patients were younger, more commonly admitted for medical reasons and had fewer comorbidities compared to those with stable housing. Processes of care in the ICU were mostly similar, but healthcare use after ICU was different; patients experiencing homelessness who survived their index hospitalization were more than twice as likely to have a visit to the emergency department (OR = 2.3 times, 95% CI 2.0–2.6, < 0.001) or be readmitted to hospital (OR = 2.1, 95% CI 1.8–2.4, *p* < 0.001) within 30 days, and stayed 10.1 days longer in hospital (95% CI 8.6–11.6, *p* < 0.001), compared with those who have stable housing.

**Conclusions:**

Patients experiencing homelessness have different characteristics at ICU admission and have similar processes of care in ICU, but their subsequent use of healthcare resources was higher than patients with stable housing. These findings can inform strategies to prepare patients experiencing homelessness for discharge from the ICU to reduce healthcare resource use after critical illness.

**Supplementary Information:**

The online version contains supplementary material available at 10.1186/s13054-023-04753-7.

## Background

Approximately 1% of the population of Organization for Economic Cooperation and Development (OECD) countries are described as homeless [1], but estimates in some countries reach as high as 8%; a number that is projected to increase [2–9]. Homelessness does not take on a singular form, but encompasses a variety of different living situations where an individual or family lacks adequate or regular housing [2–4, 10, 11]. Homelessness can be chronic (long term), cyclical (alternating between homeless and housed) or temporary (often a result of uncontrollable circumstances) [3]. Homelessness is individualistic and complex, but some factors are commonly linked with homelessness; individuals that are marginalized, have a mental illness or cognitive or physical disabilities and have history of abuse/trauma are more likely to experience homelessness [3, 12–15].

Morbidity and mortality are higher among individuals experiencing homelessness than those with stable housing, perhaps resulting from health being relegated as a lower priority than access to food, shelter and safety [12, 16–20]. Individuals experiencing homelessness have more hospital admissions than the general population and have an increased risk of acute (26% more than the general population) and chronic health conditions (46% more than the general population) [21, 22]. The severity of these health conditions is often exacerbated by uncertain living conditions, complex healthcare needs, and access to and seeking of safe health care [23].

Hospital admissions for patients experiencing homelessness on average last five times longer than low-income patients [24, 25]. One study found that ICU hospital days, acute care days and costs were substantially greater among the homeless population compared to the stable housed population [26]. Approximately one in every three hospitalizations of patients experiencing homelessness had an intensive care unit (ICU) admission, which is much greater than the rate of ICU admission among those with stable housing [22, 27–29]. Unfortunately, there is a paucity of evidence describing ICU care (e.g., use of life sustaining therapies) and outcomes (in hospital and following hospital discharge) among critically ill patients experiencing homelessness and how they compare to patients with stable housing [20, 22, 30]. Therefore, the objective of our study was to explore the care of critically ill patients experiencing homelessness compared to critically ill patients with stable housing. Specifically, to understand: What proportion of patients admitted to ICUs were experiencing homelessness and if there are differences in demographic characteristics, processes of care and outcomes between ICU patients experiencing homelessness and those with stable housing?

## Methods

### Population and setting

This is a retrospective population-based cohort study of adults (≥ 18 years old) admitted to any of the 31 ICUs within 14 hospitals in the province of Alberta, Canada, between January 2015 and April 2018. During the study period, Alberta had a population of about 4.4 million. All ICUs in Alberta are governed by a single healthcare service provider, Alberta Health Services, within a publicly funded healthcare system. All patients admitted to any ICU (general, cardiac, cardiovascular or neurosciences) in Alberta, as indicated by an ICU electronic medical record, were included unless their ICU admission lasted less than 24 h, and they were not residents of Alberta (have a primary healthcare number from another province) or did not have follow-up data for at least 180 days after index hospital discharge.

Patients were excluded if they were not residents of Alberta because our case definition of individuals experiencing homelessness required Alberta postal codes. Homelessness is complex, nuanced and fluid; however, for the purposes of analyzing quantitative data, in this study individuals were considered to be experiencing homelessness if, during any of their hospital admissions during the study period, they did not have a fixed address (had postal code T1T1T1, which is used to identify individuals experiencing homelessness, had an International Classification of Disease, 10th revision (Canadian edition; ICD-10CA) code Z59.0 indicating the patient is experiencing homelessness) or had precarious housing (their postal code was that of established housing shelters in Alberta). This definition was adopted from previous work; a case definition using postal code-based (including those of shelters) algorithms found this method was 33% sensitive and 99% specific [31], while a more recent study also included ICD-10CA codes to increase sensitivity [32].

### Data sources

All data used in this study were previously collected for administrative and clinical purposes by the data custodian, Alberta Health Services. Our cohort was identified using eCritical Alberta, a clinical information system that captures and delivers multimodal patient data to the bedside and is a repository for these data. These data include patient demographic, clinical and outcome data [33–35]. There are continuous data quality auditing to ensure data from eCritical TRACER is valid. [33]

The cohort was deterministically linked to additional data sources using a unique personal healthcare number and date of birth. These additional data sources were used to create a complete profile of participants during their hospital admission and after any hospital discharge that was associated with the ICU admission. These data sources included:

1. The Discharge Abstract Database (DAD) contains demographic, diagnostic (up to 25 International Classification of Disease version 10, Canadian codes; ICD-10-CA, with an associated diagnosis type) [36], administrative and procedural data on patients discharged from the hospital.
2. The National Ambulatory Care Reporting System (NACRS) collects and stores demographic, administrative, clinical and service-specific data from emergency departments (and other ambulatory care visits) including a complaint lists and emergency department discharge diagnoses using the Canadian Emergency Department Diagnoses Shortlist (800 diagnoses) mapped to ICD-10 codes are also collected [37].

### Variables

The exposure variable was housing status (experiencing homelessness versus stable housing) using the definitions outlined above. The proportion of patients experiencing homelessness were measured across the study period (quarters: 1 = January to March, 2 = April to June, 3 = July to September, 4 = October to December) to assess temporal trends.

The primary outcome was healthcare resource utilization. In the absence of a single measure of healthcare resource utilization, several individual variables were used to measure healthcare resource utilization: (1) processes of care, (2) ICU and hospital length of stay (number of days or part of day from admission to discharge), (3) hospital readmission (binary variable within 30 days of hospital discharge) and (4) emergency room visits (within 30 days of hospital discharge). Processes of care in the ICU included: receipt of advanced life support interventions including invasive and noninvasive mechanical ventilation, vasoactive medications and continuous renal replacement therapy (CRRT), which were binary variables (received intervention or not) and time on advanced life support were continuous variables measured in minutes or hours for those receiving the interventions. While readmission variables were calculated using all available data, only data from the index ICU and hospital admission were used to calculate lengths of stay and processes of care variables.

The secondary variables were hospital adverse events, ICU mortality and hospital mortality. Hospital adverse events were measured using validated ICD-10-CA algorithms to identify 18 patient safety indicators [38]. Each of the 18 patient safety indicators was dichotomous—ever having a hospital adverse event or not—and the overall adverse event variable was created to dichotomously indicate the presence of any adverse event.

Patient variables included age (continuous variable), sex (dichotomized as male or female), ICU admission diagnosis (categorized as medical, surgical, neurological, trauma, cardiac surgical or non-surgical or unknown) and Charlson comorbidities (categorized as none, one comorbidity or two or more comorbidities). The reason for hospital admission (the most responsible diagnosis as defined by diagnosis type and categorized by the ICD-10-CA chapters) and any mental health-specific diagnosis related to the hospital admission (ICD-10-CA codes for depression, anxiety, substance use, psychosis, suicide or severe psychiatric disorders that were noted as the reason for hospital admission, comorbidities or that occurred in hospital) were identified.

### Statistical analysis

The demographic characteristics of the cohort were explored using descriptive statistics. The exposure variable, patients experiencing homelessness, was described as a frequency with proportion. Seasonal and temporal trends in homelessness were explored using quarters (Q1 = January to March, Q2 = April to June, Q3 = July to September, Q4 = October to December). The demographic characteristics, healthcare resource utilization and outcomes of patients experiencing homelessness and those who had stable housing were compared using Chi-squared tests for categorical variables and Wilcoxon rank-sum tests for continuous variables.

Regression models (linear for continuous outcome variables and logistic for dichotomous outcome variables) were developed to address each of the research questions. Pre-specified patient variables were included in multivariable regression models to control for potential effect measure modifiers and/or confounders. Specifically, age, sex, Charlson comorbidity index and type of ICU admission were included in each model.

Data analysis was conducted using Stata version 17 [39]. Statistical significance was set at α = 0.05 for both univariate analysis and multivariable regression.

## Results

### Participants

**Fig. 1:**
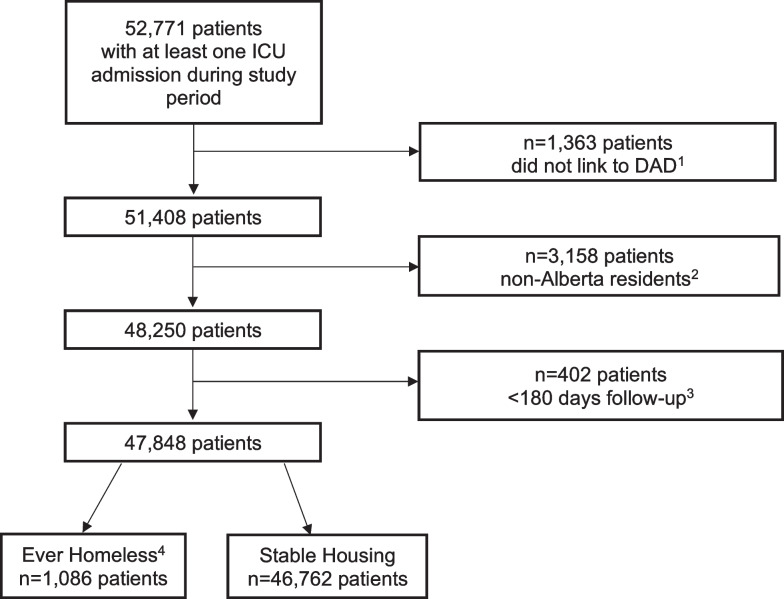
Study cohort diagram

During the study period, there were 52,771 patients with at least one ICU admission among 47,848 unique patients; 2.3% (1086) of patients experienced homelessness and 97.7% (46,762) had stable housing at the time of admission during the study period (Fig. 1). Of those experiencing homelessness, 37.7% (*n* = 409) were identified using postal codes and 97.4% (*n* = 1058) were identified using ICD-10 codes. (Percent is greater than 100 because 127 patients were identified using both methods.) The proportion of patients admitted to the ICU experiencing homelessness was consistent across the study period (range = 2.2–3.2%; Chi-squared = 16.8, *p* = 0.156), but did vary by hospital site (range = 0.5% to 4.2%; Chi-squared = 337.0, *p* < 0.001).

The characteristics of the study population are described in detail in Table 1. The majority of patients were male (63.4%) with a median age of 62 (IQR = 52–73) years, admitted for a non-surgical cardiac reason (40.4%) and had multimorbidity. ICU patients experiencing homelessness were younger, more commonly admitted for medical reasons and had fewer comorbidities compared to those who had stable housing (Table 1).

**Table 1:** Demographic information

Variable	Cohort (*n* = 47,848)	Ever homeless (*n* = 1086)	Stable housing (*n* = 46,762)	*p* values
Basic demographics				
Age, median (IQR)	62 (52, 73)	50 (39, 59)	63 (52, 73)	< 0.001
Male, *n* (%)	30,316 (63.4)	744 (68.5)	29,572 (63.2)	< 0.001
ICU admission diagnosis, *n* (%)				
Medical	12,901 (27.0)	646 (59.5)	12,255 (26.2)	< 0.001
Surgical	2269 (4.7)	76 (7.0)	2193 (4.7)	
Neurological	5275 (11.0)	82 (7.6)	5193 (11.1)	
Trauma	1434 (3.0)	89 (8.2)	1345 (2.9)	
Cardiac: non-surgical (CCU)	19,309 (40.4)	149 (13.7)	19,160 (41.0)	
Cardiac: surgical (CVICU)	5733 (12.0)	27 (2.5)	5706 (12.2)	
Unknown	927 (1.9)	17 (1.6)	910 (1.9)	
Comorbidities, *n* (%)^a^				
Diabetes	12,758 (26.7)	242 (22.3)	12,516 (26.8)	< 0.001
Chronic lung disease	4682 (9.8)	137 (12.6)	4545 (9.7)	0.001
Chronic kidney disease	1910 (4.0)	31 (2.9)	1879 (4.0)	0.053
Liver disease	1998 (4.2)	134 (12.3)	1864 (4.0)	< 0.001
Cancer	3533 (7.4)	22 (2.0)	3511 (7.5)	< 0.001
Chronic heart or peripheral vascular disease	19,136 (40.0)	215 (19.8)	18,921 (40.5)	< 0.001
Neurological disease	3207 (6.7)	67 (6.2)	3140 (6.7)	0.48
Comorbidity scores				
Charlson 0, *n* (%)	14,196 (29.7)	477 (43.9)	13,719 (29.3)	< 0.001
Charlson 1, *n* (%)	14,531 (30.4)	266 (24.5)	14,265 (30.5)	
Charlson ≥ 2, *n* (%)	19,121 (40.0)	343 (31.6)	18,778 (40.2)	
APACHE II on admission, median (IQR)*	17 (12–24)	17 (12–24)	17 (12–24)	0.59

*APACHE II had missing data (mostly for patients in the coronary care units); cohort *n* = 27,789, ever homeless *n* = 922, stable housing *n* = 26,867

^a^Comorbidities recorded in eCritical

**Fig. 2:**
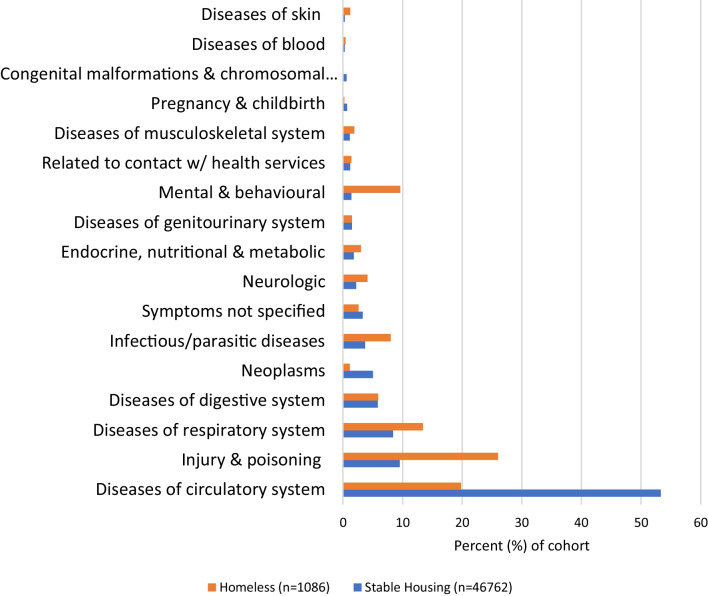
Reason for hospital admission

The reason patients were admitted to hospital differed between those who were experiencing homelessness and those who had stable housing (Additional file 1: Table S1, Fig. 2). ICU patients who were experiencing homelessness were more commonly admitted with a mental health diagnosis (depression, anxiety, substance use disorder, psychosis, suicide and severe psychiatric disorder). The psychological diagnosis with the largest difference between patients experiencing homelessness and those with stable housing was substance use disorder (44.6% vs. 7.5%, *p* < 0.001) followed by attempted suicide (21.5% vs. 4.0%, *p* < 0.001). When examining other reasons for admission to the hospital, patients experiencing homelessness most commonly were admitted due to “injury, poisoning and consequences of external causes” followed by “diseases of the circulatory system” and “diseases of the respiratory system,” whereas the majority of those who had stable housing were admitted for “diseases of the circulatory system” (Fig. 2).

### Primary outcome (healthcare resource utilization)

On nearly every process of care measure, patients experiencing homelessness received more life support interventions than patients who had stable housing; however, after controlling for age, sex, comorbidity and ICU admission diagnosis there were few differences between ICU patients who were experiencing homelessness and those who had stable housing (Table 2). Patients experiencing homelessness were more likely to receive invasive mechanical ventilation, but there were no differences in the proportion of patients receiving noninvasive mechanical ventilation, vasoactive medications or continuous renal replacement therapy (Table 2).

**Table 2:** Processes, healthcare resource utilization and outcomes of care

Variable	Cohort (*n* = 47,848)	Homeless (*n* = 1086)	Stable Housing (*n* = 46,762)	Crude OR/mean difference (95% CI)	Adjusted OR/mean difference^a^ (95% CI)
Processes of care, *n* (%)					
Invasive ventilation *n* (%)	20,471 (42.4)	695 (63.0)	19,776 (41.9)	2.3 (2.1 to 2.6)	1.5 (1.3 to 1.8)
Invasive ventilation median hours (IQR)*	23.4 (8.2–80.6)	40.1 (16.5–116.6)	22.9 (7.9–79.0)	23.0 (10.9 to 35.2)	0.9 (− 11.1 to 12.9)
Noninvasive ventilation *n* (%)	3613 (7.5)	76 (6.9)	3537 (7.5)	0.9 (0.7 to 1.2)	0.7 (0.6 to 0.9)
Noninvasive ventilation, median hours (IQR)*	17.2 (6.3–46.9)	21.2 (5.2–55.6)	17.2 (6.3–46.7)	− 0.3 (− 16.3 to 15.8)	− 2.5 (− 18.6 to 13.5)
Vasoactive medications *n* (%)	16,531 (34.3)	414 (37.5)	16,117 (34.2)	0.1 (0.0 to 0.3)	1.0 (0.9 to 1.1)
Vasoactive medications median minutes (IQR)*	1419.0 (596.0–2915.0)	1647.0 (811.0–3440.0)	1416.5 (591.5–2899.5)	92.7 (− 103.3 to 288.6)	− 127.4 (− 321.9 to 67.2)
CRRT *n* (%)	1335 (2.8)	38 (3.4)	1297 (2.8)	1.3 (0.9 to 1.8)	0.7 (0.5 to 1.0)
CRRT Median hours (IQR)*	65.4 (27.0–140.1)	85.6 (24.1–141.4)	65.3 (27.1–140.0)	− 5.4 (− 52.5 to 41.6)	− 5.7 (− 53.0 to 41.6)
Healthcare resource utilization, *n* (%)					
Length of ICU stay, median (IQR)	2.7 (1.4–4.8)	3.4 (1.7–6.7)	2.6 (1.4–4.8)	1.5 (1.1 to 1.9)	0.5 (0.1 to 0.9)
Length of hospital stay, median (IQR)	6.6 (3.3–14.0)	9.9 (4.2–25.0)	6.5 (3.3–14.0)	12.8 (11.3 to 14.4)	10.1 (8.6 to 11.6)
ED visit within 30 days post-hospital discharge	10,957 (22.9)	401 (36.9)	10,556 (22.6)	2.0 (1.8 to 2.3)	2.3 (2.0 to 2.6)
Hospital readmission within 30 days post-hospital discharge	7693 (16.1)	293 (27.0)	7400 (15.8)	2.0 (1.7 to 2.3)	2.1 (1.8 to 2.4)
Safety of care outcomes					
Hospital adverse event *n* (%)	11,392 (23.8)	271 (25.0)	11,121 (23.8)	1.1 (0.9 to 1.2)	1.1 (0.9 to 1.2)
ICU mortality *n* (%)	3700 (7.7)	60 (5.5)	3640 (7.8)	0.7 (0.5 to 0.9)	0.5 (0.4 to 0.6)
Hospital mortality *n* (%)	5235 (10.9)	89 (8.2)	5146 (11.0)	0.7 (0.6 to 0.9)	0.5 (0.4 to 0.7)

*CRRT* continuous renal replacement therapy, *ED* emergency department, *IQR* interquartile range, *OR* odds ratio, *95% CI* 95% confidence interval

*Among those that had the intervention

^a^Adjusted for sex, age and Charlson comorbidity index (category) and ICU admission diagnosis

With regard to healthcare resource utilization, patients experiencing homelessness stayed on average 0.5 days longer in the ICU and 10.1 days longer in hospital than patients who had stable housing (Table 2). Similarly, patients who were experiencing homelessness who survived their index hospitalization were twice as likely to have a visit to the emergency department or be readmitted to hospital within 30 days of their index hospital discharge compared with those who have stable housing (Table 2).

### Secondary outcomes (patient safety and hospital and ICU mortality)

**Fig. 3:**
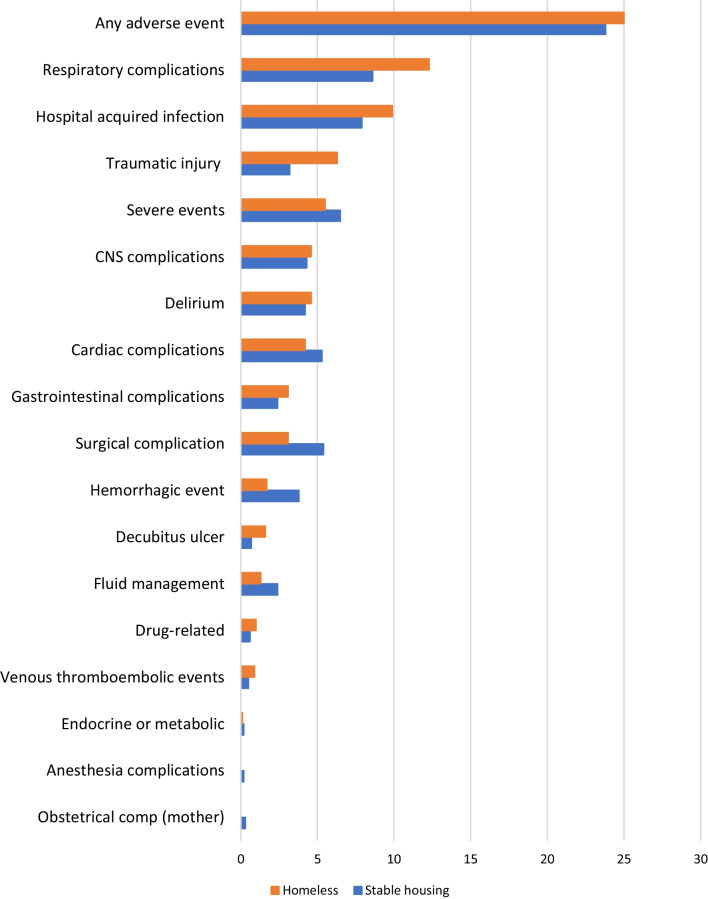
Patient safety events

There were no differences in the proportion of patients who experienced a hospital adverse event between patients experiencing homelessness and those who had stable housing (Table 2). There were differences in the types of adverse events. Patients experiencing homelessness were more likely to have respiratory complications and traumatic injury during the hospital stay and less likely to have hemorrhagic complications compared with patients who had stable housing (Fig. 3). Patients experiencing homelessness were less likely to die in the ICU or in the hospital (Table 2).

## Discussion

This study found that one in 43 patients admitted to the ICU was experiencing homelessness (2.3% of ICU admissions). Patients experiencing homelessness differ on baseline characteristics compared to patients who had stable housing; they were younger, had fewer comorbidities and were admitted for medical reasons such as a mental health related diagnosis (e.g., substance use disorder and attempted suicide). Despite the smaller portion of ICU admissions who were experiencing homelessness, these patients used more healthcare resources, especially after their index ICU admission. Despite differences in baseline characteristics and healthcare resource use, patients experiencing homelessness were less likely to die in ICU or hospital.

There is a sparsity of studies examining critical illness among patients experiencing homelessness and comparing critically ill patients experiencing homelessness to patients with stable housing [30]. One study estimated 0.3% of critically ill patients were experiencing homelessness—the estimate in the present study is considerably greater with 2.3% of ICU patients experiencing homelessness [27]. The reason for differences between studies may reflect heterogeneous rates of homelessness across countries and jurisdictions. Indeed, we found variation in the proportion of patients experiencing homelessness across our ICU sites highlighting the importance of using population-based data. Differences in estimates between studies may also be related to the case definition of homelessness. The validated case definition used in this study was tailored to the province of Alberta by including postal codes for local shelters [31, 32], whereas the lower estimate of ICU patients experiencing homelessness in the study by Nathanson et al. [27] may be related to the use of only ICD-9 codes to identify patients experiencing homelessness. Indeed, using the postal codes of homeless shelters in the province, we identified 14.3% of the cohort of patients experiencing homelessness defined by temporarily residing in these shelters (which could be classified as precarious housing). Identifying and classifying patients experiencing homelessness can be challenging, especially given varying degrees of housing security and the fluidity of homelessness and can lead to variation between studies. Nevertheless, our study suggests that this is common among patients admitted to ICU and may even underestimate the number of patients experiencing homelessness given our case definition is reported to have high specificity with lower sensitivity. Further studies exploring generalizable approaches for identifying patients experiencing homelessness will help advance our understanding of healthcare use and outcomes for this vulnerable population.

Baseline characteristics are often associated with differences in healthcare resource use and outcomes. This study found differences in baseline characteristics, including comorbid conditions and reasons for admission to hospital and ICU between patients experiencing homelessness and those with stable housing. Some conditions may disproportionately affect individuals experiencing homelessness which include cancer, musculoskeletal disorders, skin ailments, severe foot conditions, pulmonary issues, infections, traumatic injuries, substance abuse disorders and mental illnesses [4, 40–42]; the findings of the present study are consistent with this. Most notably, patients experiencing homelessness were more commonly admitted with a mental health related diagnosis than patients with stable housing, especially substance use disorder and attempted suicide. It has been estimated that substance use accounts for a quarter of ICU resource utilization among patients medical ICU admissions [43]. The relationship between mental health and healthcare resource use could not be explicitly explored in detail in this study, but is an interesting topic for future research. Despite differences in underlying conditions and reasons for admission, our study found that patients experiencing homelessness did not differ from patients who had stable housing on the APACHE II score on admission to the ICU (should be interpreted with caution as this excludes patients admitted to cardiac care units), and they had fewer documented comorbidities and had similar processes of care in the ICU. This finding challenges the hypothesis that individuals experiencing homelessness neglect their health and/or wait too long to seek help [12, 16–20], but prospective studies that explore this relationship are needed.

This study also found that resource use differed after the index ICU admission, suggesting possible differences in clinical course between patients experiencing homelessness compared to patients who had stable housing. This is supported by the higher healthcare resource use during and after critical illness among patients experiencing homelessness compared to patients with stable housing. The findings of this study are consistent with those of others that have found consistently high resource use among patients experiencing homelessness and add evidence to suggest the higher resource utilization extends beyond hospital length of stay and emergency department visits to critical care. Those experiencing homelessness have been found to use a high number of emergency services with 80% of these visits related to illnesses that would be suitable for management utilizing preventative care [41, 44–46]. It has been found that patients experiencing homelessness have heightened care intensity and longer hospital stays resulting in an increased cost of $2559–$2907 per hospital admission when compared to patients that are stably housed of similar age and sex [4, 22, 25, 26, 47]. In addition to increased use of emergency department and acute care, there exists a high utilization of critical care resources among those experiencing homelessness [23], which is supported by the finding that patients experiencing homelessness used more invasive mechanical ventilation. However, the resource intensity of patients experiencing homelessness during their ICU and hospital stay may have been appropriately necessary—patients experiencing homelessness did not die any more often in ICU or hospital due to their critical illness. The findings extend the current understanding of healthcare resource use, specifically what ICU resources are consumed by patients experiencing homelessness, which can help healthcare providers and healthcare systems plan resources required to treat patients experiencing homelessness in ICUs and as they transition from the ICU to the hospital or discharged to the community. Care pathways and discharge planning has been a successful strategy for managing discharge among the general critical care population and maybe present an opportunity to improve the transition out of the ICU for patients experiencing homelessness [35, 48, 49]. Future research is needed to explore the potential effectiveness of these strategies, which perhaps need to be initiated earlier among patients experiencing homelessness given the complexity of their situations.

This study has several strengths; namely, we were able to leverage population-based administrative and clinical data to explore trends in critical illness and the outcomes of critical illness among patients experiencing homelessness across 31 ICUs and we used a validated case definition to identify the cohort, but there are limitations that should be considered when interpreting our findings. While we used a validated case definition of homelessness [31, 32], it is important to note that homelessness is a complex and fluid concept that is challenging to simplify into a binary variable. To this end, the validated case definition we used was specific, but not very sensitive suggesting that we may have misclassified some patients as having stable housing when in fact they were experiencing homelessness. While these population-based data were a strength of this study, the retrospective analysis of these data precluded the evaluation of the complex relationship between mental health and outcomes. Finally, the data represent a cohort before the COVID-19 pandemic and it is unclear whether incidence and consequences of homeless have changed.

## Conclusion

To conclude, those experiencing homelessness are admitted to ICUs regularly. Their course in the ICU was broadly similar to patients who have stable housing, but their use of subsequent healthcare resources was different; they were more likely to stay in hospital longer, have more emergency department visits and be readmitted to hospital than patient with stable housing. These findings suggest that patients experiencing homelessness are receiving similar care while in the ICU, but that their health journey after discharge from the ICU differs from patients with stable housing. There is a clear need to identify strategies to support the long-term health of patients experiencing homelessness as they recover from critical illness. The findings of this study can inform the development, implementation and evaluation of such strategies and can advocate for resources for early ICU discharge planning to address the unique needs of critically ill patients experiencing homelessness.

### Supplementary Information

**Additional file 1. Supplemental Table 1.** Diagnostic Information.

## Data Availability

Administrative data and clinical data used in this study are available upon reasonable request from the corresponding author.
