# Supplementary material for: Critical illness among patients experiencing homelessness: a retrospective cohort study
Source: Crit Care. 2023 Dec 6;27:477. doi: 10.1186/s13054-023-04753-7 (PMC10699027; doi:10.1186/s13054-023-04753-7)
Supplement: Supplementary file 1 — Additional file 1. Supplemental Table 1. Diagnostic Information. [file 13054_2023_4753_MOESM1_ESM.docx]

**Supplemental Table 1.** Diagnostic Information

| **Variable** | **Cohort (n=47848)** | **Homeless (n=1086)** | **Stable Housing**  **(n=46762)** | **p values** |
| --- | --- | --- | --- | --- |
| Infectious and parasitic diseases | 1795 (3.8) | 87 (8.0) | 1708 (3.7) | <0.001 |
| Neoplasms | 2356 (4.9) | 12 (1.1) | 2344 (5.0) |  |
| Diseases of blood and blood forming organs | 125 (0.3) | 4 (0.4) | 121 (0.3) |  |
| Endocrine, nutritional and metabolic diseases | 882 (1.8) | 33 (3.0) | 849 (1.8) |  |
| Mental and behavioural | 750 (1.6) | 104 (9.6) | 646 (1.4) |  |
| Diseases of the nervous system | 1085 (2.3) | 44 (4.1) | 1041 (2.2) |  |
| Diseases of the eye and adnexa | 4 (0.0) | 0 (0.0) | 4 (0.0) |  |
| Diseases of the ear and mastoid process | 6 (0.0) | 0 (0.0) | 6 (0.0) |  |
| Diseases of the circulatory system | 25118 (52.5) | 215 (19.8) | 24903 (53.3) |  |
| Diseases of the respiratory system | 4056 (8.5) | 146 (13.4) | 3910 (8.4) |  |
| Diseases of the digestive system | 2793 (5.8) | 64 (5.9) | 2729 (5.8) |  |
| Diseases of the skin and subcutaneous tissue | 152 (0.3) | 13 (1.2) | 139 (0.3) |  |
| Diseases of the musculoskeletal system and connective tissue | 540 (1.1) | 21 (1.9) | 519 (1.1) |  |
| Diseases of the genitourinary system | 702 (1.5) | 16 (1.5) | 686 (1.5) |  |
| Pregnancy, childbirth and the puerperium | 313 (0.7) | 2 (0.2) | 311 (0.7) |  |
| Congenital malformations, deformations, and chromosomal abnormalities | 295 (0.6) | 0 (0.0) | 295 (0.6) |  |
| Symptoms, signs and abnormal clinical and laboratory findings, not elsewhere classified | 1554 (3.2) | 28 (2.6) | 1526 (3.3) |  |
| Injury, poisoning and certain consequences of external causes | 4725 (9.9) | 282 (26.0) | 4443 (9.5) |  |
| Factors influencing health status and contact with health services | 597 (1.2) | 15 (1.4) | 582 (1.2) |  |
| Mental Health Specific Diagnoses |  |  |  |  |
| Depression | 1613 (3.4) | 123 (11.3) | 1490 (3.2) | <0.001 |
| Anxiety | 737 (1.5) | 36 (3.3) | 701 (1.5) | <0.001 |
| Substance Use Disorder | 4003 (8.4) | 484 (44.6) | 3519 (7.5) | <0.001 |
| Psychosis | 363 (0.8) | 42 (3.9) | 321 (0.7) | <0.001 |
| Suicide | 2108 (4.4) | 233 (21.5) | 1875 (4.0) | <0.001 |
| Severe Psychiatric Disorder | 310 (0.6) | 30 (2.8) | 280 (0.6) | <0.001 |
